# The Role of Ammonia-Oxidizing Archaea During Cycling and Animal Introduction in a Newly Commissioned Saltwater Aquarium

**DOI:** 10.3390/ani15101446

**Published:** 2025-05-16

**Authors:** Francis J. Oliaro, Oluwaseun Ajileye, Iris George, Sal Lamsal, Ilana A. Mosley, Bradly Ramirez, Tiana L. Sanders, Veerakit Vanitshavit, William Van Bonn, Lee J. Pinnell

**Affiliations:** 1 Animal Care and Science Division, John G. Shedd Aquarium, Chicago, IL 60605, USA; foliaro@sheddaquarium.org (F.J.O.); billvanbonn@gmail.com (W.V.B.); 2 Ecology and Evolutionary Biology Program, Texas A&M University, College Station, TX 77843, USA; ajileye_.5@tamu.edu; 3 Department of Veterinary Pathobiology, Texas A&M University, College Station, TX 77843, USA; iris.2022@tamu.edu (I.G.); tlsanders2415@tamu.edu (T.L.S.); veerakit@tamu.edu (V.V.); 4 Veterinary Education, Research, and Outreach Program, Texas A&M University, Canyon, TX 79015, USA; sal@tamu.edu (S.L.); ramirez_b@tamu.edu (B.R.); 5 Department of Veterinary Integrative Biosciences, Texas A&M University, College Station, TX 77843, USA; ixm57@tamu.edu

**Keywords:** ammonia-oxidizing archaea, nitrification, aquaria, host-associated microbiome

## Abstract

Recirculating saltwater aquarium exhibits and systems must be meticulously designed and maintained to support the life of not only resident animals but also stable communities of beneficial microbes that process nitrogenous waste. These nitrifying microbial communities act as biofilters that are critical to system functioning, yet little is known about succession dynamics in system startup and eventual impacts of host-associated microbiota on the aquarium environment. Here, we characterized microbial community dynamics in a large public saltwater aquarium over a 54-day period spanning nitrogen cycling, the addition of live foods (*Artermia franciscana* and *Americamysis bahia*), and the introduction of weedy seadragons and sea urchins. Daily water samples were collected for 16S rRNA amplicon sequencing, and routine measurements of temperature, pH, salinity, and nitrogen species (ammonium, nitrite, nitrate) were performed. Results revealed that ammonia-oxidizing archaea (AOA), particularly *Nitrosopumilus*, were the dominant nitrifiers during startup, peaking in abundance during periods of high ammonium additions. Alpha and beta diversity analyses showed significant changes in microbial community structure following the introduction of live animals, with the seadragon phase exhibiting the most distinct community composition. These findings underscore the importance of AOA in saltwater aquarium nitrification and demonstrate the strong influence of host-associated microbial inputs on microbial succession in closed aquatic systems.

## Simple Summary

Saltwater aquarium systems rely on microbial communities to help maintain water quality, especially during the critical startup phase of new exhibits. These microbes convert nitrogenous waste—produced by feeding and animal metabolism—into less harmful forms, a process known as nitrogen cycling. However, little is known about how these microbial communities change over time or respond to the introduction of animals and live foods. In this study, we monitored daily microbial community composition and water quality parameters in a newly commissioned public saltwater aquarium over 54 days. We observed that ammonia-oxidizing archaea were the primary microbes responsible for early nitrogen cycling. Once live foods and weedy seadragons were introduced, microbial communities shifted substantially in both composition and diversity. These results demonstrate that animal additions can rapidly alter microbial dynamics, even in well-established systems. Understanding these changes can help aquariums more effectively manage new exhibits by informing the timing of biological inputs and ensuring stable water conditions for animal health and welfare.

## 1. Introduction

Closed recirculating aquatic systems (aquariums) offer a multitude of benefits including the ability to observe and research aquatic animals ex situ, and under controlled environmental conditions [1]. Whether it be for purposes of aquaculture, private, or public display, a suitable, healthy environment for the inhabitants of the system is paramount. Nitrogen is a vital nutrient for living organisms; however, an excess of reactive nitrogen species, such as ammonia, nitrite, and nitrate, can lead to eutrophication, harmful algal blooms, and oxygen depletion in aquatic systems [2,3]. Open-system aquariums benefit from direct connection to a natural body of water that offers constant circulation, thus minimizing ammonia concentrations [4]. In closed systems, ammonia excreted by fishes, primarily through their gills, and ammonification of organic nitrogenous compounds such as feces and uneaten food [5] accumulates over time. Water quality in a closed system can quickly become dangerous to resident animals without continuous conversion of ammonia to less harmful compounds. Ammonia is toxic to marine animals in concentrations as low as 0.2 ppm [6], and high ammonia concentrations in aquariums can cause damage to the animals’ tissue and internal organs and become lethal [7,8]. Ammonia accumulation can trigger oxidative stress, tissue damage, and disrupted gene expression in fish, with effects exacerbated by environmental stressors such as salinity and elevated temperature [9,10]. More broadly, ammonia also plays a physiological role in fish and its environmental buildup poses significant risks to recirculating aquatic ecosystems [11].

The critical importance of biological filtration in recirculating aquatic systems is widely recognized among aquarium professionals, hobbyists, and managers of aquaculture facilities [12]. This is achieved through the natural process of nitrification, by which ammonia (NH_3_) is oxidized to nitrite (NO_2_) followed by oxidation of nitrite to nitrate (NO_3_). Nitrifying microbial communities living within filter media, substrate, and water of the aquarium system initiate and control nitrification, thus maintaining system homeostasis. Nitrification is a stepwise process driven by chemolithoautotrophic ammonia-oxidizing bacteria (AOB), e.g., *Nitrosomonas* and *Nitrosococcus* spp. [13]; ammonia-oxidizing archaea (AOA), e.g., *Nitrosopumilus* spp. [14]; and nitrite-oxidizing bacteria (NOB), e.g., *Nitrobacter* and *Nitrospira* spp. [15]. Organisms (e.g., *Nitrospira* spp.) capable of performing complete oxidation of ammonia to nitrate (commamox) have been characterized [15] and were found to be highly abundant in freshwater aquaria, but not in saltwater aquaria [16].

Stable and adequate biofiltration via nitrification is a prerequisite to animals being added to an aquarium system, and thus it is of great interest to system operators to establish a population of nitrifying organisms quickly and effectively. Unaided, it can take many weeks for a stable nitrifying microbial community to establish in an aquarium, and for water ammonia and nitrite levels to decrease safe levels. As such, there are many common practices used by system managers designed to quickly initiate or improve nitrogen cycling by “seeding” a system with nitrifying communities. This could be in the form of adding “live rock”, filter media, or substrate taken from other aquaria already established with nitrifying communities; introduction of fish food; dosing of 100% ammonia; or by adding commercially available products containing nitrifying bacteria [17,18,19]. Although these practices are routinely used, little is known about their efficacy or the succession of microbial taxa in a system as nitrification is established. Recent studies have documented shifts in saltwater aquarium microbiota in response to perturbations such as large volume water changes [20,21], and addition of live rock to closed seawater systems [22]. All studies found significant changes to composition of microbial communities, including increased proportions of nitrifying taxa as well as changes in community diversity after the system perturbations. Patin et al. [23] examined a longitudinal stability in an established, large-volume, artificial seawater system and found dynamic fluctuations of the water column microbiome associated with bloom events and increases in turbidity.

The present study aimed to describe the temporal succession of microbial communities in a public saltwater aquarium during recommissioning and early biological loading, subsequent periods of seeding, introduction of live foods (*Artermia franciscana* and *Americamysis bahia*), and resident weedy seadragons (*Phyllopteryx taeniolatus*) and thorny sea urchins (*Prionocidaris* sp.). Specifically, our objective was to characterize how routine management activities and the introduction of animals influence microbial diversity and composition in a newly commissions closed saltwater aquarium. We hypothesized that changes in microbial diversity and composition would correlate with changes in environmental conditions (i.e., temperature, pH, NH_3_, etc.) due to management decisions (i.e., filter backwashes, water additions, etc.) and that microbial diversity and composition would change dramatically following the introduction of host-associated microbiota from live foods and resident species.

Further, we hypothesized that AOB would be primarily responsible for ammonia oxidation, as described previously. To test these hypotheses, we employed 16S rRNA amplicon sequencing to assess the diversity and composition of the overall microbial community, with a specific focus on ammonia-oxidizing microbes, over the first 54 days of commissioning a new saltwater aquarium. This timeline encompassed the initial nitrogen cycling period; the introduction of nauplii and mysids; and, ultimately, the presence of nauplii, mysids, sea urchins, and weedy seadragons.

## 2. Methods

### 2.1. Aquarium System and Water Sampling

A large saltwater exhibit located at John G. Shedd Aquarium, Chicago, IL, USA, was used as the study system after a decommission was planned for in early 2016. The total system volume is 5000 gallons, which is sourced from the municipal supply, filtered through activated carbon, and mixed with a commercial sea salt designed for aquarium maintenance (Instant Ocean, Blacksburg, VA, USA). Water is recirculated through the exhibit life support system consisting of mechanical (sand) filters, a biofiltration tower containing media to maximize microbial biofilm surface area, foam fractionation, and a side stream UV disinfection loop. Routine water quality measurements of temperature, pH, salinity, ammonia (NH_3_), nitrite (NO_2_), and nitrate (NO_3_) were taken approximately once weekly in the year prior to and then daily for the week leading up to decommission of the system. pH, salinity, and temperature were measured using benchtop probes (pH) and handheld meters (salinity, temperature). Colorimetric benchtop assays and a spectrophotometer (Hach Company, Loveland, CO, USA) were used to measure ammonia, nitrite, and nitrate. The mean values during that period are as follows: temperature (63.0 °F), pH (8.24), salinity (33.9 ppt), ammonia (0.002 ppm), nitrite (0.006 ppm), nitrate (3.17 ppm). Prior to the shut-down, the exhibit housed weedy seadragons (*Phyllopteryx taeniolatus*). Live nauplii and mysid cultures were added to the exhibit as food for the seadragons. For detailed information about water quality measures on each sampling day, please see the metadata table in the associated GitHub repository (https://github.com/ljpinnell/Aquarium_Nitrogen_Seadragons) (accessed on 29 April 2025).

Based upon findings of potential protozoal infection made during clinical diagnostics of seadragons from the exhibit, the decision was made to move all animals to a reserve system for treatment and to disinfect the exhibit system. This provided the opportunity examine the succession of environmental microbial communities as the exhibit was recommissioned. Sodium percarbonate at a final concentration of 150 ppm was used to disinfect the system. After disinfection, the system was rinsed and drained. The gravel substrate from the exhibit tank was then replaced with aragonite sand (CaribSea, Fort Pierce, FL, USA) and rinsed to drain on 3 March 2016. On 7 March 2016, approximately 400 gallons of water was added to the 750-gallon sump along with a squid and fish slurry to initiate the process of ammonia oxidation and our daily sampling began. Only the sump contained water until 12 April 2016 (day 36 of study), when water was added to the exhibit tank and began circulating with the sump. On 13 April 2016 (day 37 of study), live food (adult mysids + nauplii) was added to the exhibit, and on 17 April 2016 (day 41 of study), 6 weedy seadragons and 4 thorny sea urchins were added to the exhibit. During daily routine sampling for water quality parameters (temperature, pH, salinity, ammonia, nitrite, nitrate), water samples were also collected for microbial community analysis. Sampling was conducted at the same time each day from 7 March 2016 through 30 April 2016, which effectively captured the time frame of seeding the system, establishing stable nitrification, addition of live foods to the system, and finally the reintroduction of the resident seadragons. During the seeding period, increasing volumes of ammonium chloride (1–25 mL) were added to the system from 16 March through 16 April. For a full description of events that occurred during the 54-day sampling period, please see Supplementary Table S1. Five hundred milliliters of water was collected from 6–12 inches below the water surface in the system sump using autoclaved Nalgene^®^ bottles. Each sample was filtered through a 0.2 μm Sterivex cartridge (Millipore Corporation, Billerica, MA, USA) within 1–2 h of sample collection using a sterile 50 mL syringe. After filtration, both ends of the Sterivex cartridge were sealed with parafilm to prevent desiccation and contamination, placed in Whirlpak^®^ (Nasco, Pleasant Prarie, WI, USA) bags, and frozen at −80 °C prior to DNA extraction approximately three to four months later.

### 2.2. DNA Extraction, PCR, and Illumina Sequencing

Genomic DNA was extracted from Sterivex cartridges using the PowerWater Sterivex DNA Isolation Kit (MO BIO, Carlsbad, CA, USA) according to the manufacturer’s suggested protocol, and they were quantified using the Qubit 3.0 fluorometer and High Sensitivity dsDNA kit (Life Technologies, Carlsbad, CA, USA). Bacterial and archaeal DNA was amplified using primer constructs targeting the V4 region of the 16S rRNA gene [24]. Constructs contain Illumina specific adapters, 12 bp Golay barcodes on each forward primer, primer pads, and linkers, followed by the template-specific PCR primer. PCR was performed in replicate 25 µL reactions containing 10 µL 2.5× 5PRIME HotMasterMix (Quantabio, Beverly, MA, USA), 0.2 µM forward primer 515f and reverse primer 806rB, 1 µL of template DNA, and nuclease-free water to equal the final reaction volume of 25 µL. Thermal cycling conditions were carried out as follows: 94 °C for 3 min, 35 cycles at 94 °C for 45 s, 50 °C for 60 s, and 72 °C for 90 s, with a final extension of 10 min at 72 °C. Replicate amplicons were combined, and 5 µL was electrophoresed in 1.8% agarose gels to confirm amplification of the V4 region. Amplicon libraries were then quantified using Quant-iT™ PicoGreen™ dsDNA Reagent (Life Technologies, Carlsbad, CA, USA) and a microplate reader. From each amplicon library, 25 µL was then cleaned and normalized using the SequalPrep™ Normalization Plate Kit (Applied Biosystems). One hundred forty nanograms of each amplicon library were pooled, and the combined library was purified using the UltraClean PCR Clean-Up Kit (MO BIO, Carlsbad, CA, USA). The molarity of the pooled library was calculated, and the pool was diluted to 2 nM before denaturation and further dilution to a loading concentration of 8 pM. Paired-end sequencing for a total of five hundred cycles was conducted on the Illumina MiSeq platform using custom sequencing primers described previously [25] with the addition of 10% PhiX Control library (Illumina, San Diego, CA, USA) to increase sequence diversity.

### 2.3. Bioinformatics

Demultiplexed paired-end reads were imported into QIIME2 version 2023.2 [26] using the ‘qiime tools import’ and ‘qiime demux summarize’ tools [27] via computing resources from Texas A&M University High Power Research Computing Facilities (TAMU HPRC). DADA2 was used to filter reads for quality, remove chimeric sequences, merge overlapping paired-end reads, and generate amplicon sequence variants (ASVs) within QIIME2 [28]. Forward and reverse reads were truncated at 249 bp and trimmed at 19 bp and 20 bp (forward and reverse reads, respectively). The resulting representative sequences of each unique ASV were used to assign taxonomy using a classifier trained on the V4 region (515f/806r) of the 16S rRNA gene based off of the SILVA reference database (SILVA release 138.1) [29] using the ‘qiime feature-classifier classify-sklearn’ tool. Reads corresponding to mitochondria and chloroplast sequences were filtered out of both the ASV count matrix and representative sequences table using the ‘qiime taxa filter-table’ and ‘qiime taxa filter-seqs’ tools, respectively. A mid-point rooted phylogenetic tree was constructed using the ‘qiime alignment mafft’, ‘qiime alignment mask’, ‘qiime phylogeny fasttree’, and ‘qiime phylogeny midpoint-root’ tools. Data were imported into R Studio 4.3.1 [30] using the ‘import_biom’ function of the phyloseq package [31].

### 2.4. Visualization and Statistical Analysis

To contextualize microbial succession over the 54-day sampling period, we divided the timeline into 11 distinct phases. These phases were delineated based on operational events and system milestones recorded by aquarium staff, including changes in temperature, saltwater and freshwater additions, the start and stop of ammonium chloride dosing, sand filter backwashing, exhibit tank filling, and the introduction of live foods and resident animals (see Table S1 for a detailed description of each phase). This classification was not data-driven but instead established a priori to align microbial community shifts with biologically or operationally meaningful transitions during system cycling and early stocking. Alpha diversity was measured using observed ASVs (richness) and Shannon’s diversity index, which were calculated using the phyloseq package. Richness and diversity were compared between phases with and without animals present. These phases represent days 1 to 36 when there were no animals present (“no animals”), day 37 to 41 when nauplii and mysids were present (“live foods”), and day 42 to 54 when weedy seadragons and thorny sea urchins were added (“seadragons”). Differences between animal phases were tested using a pairwise Wilcoxon rank-sum ANOVA with a Benjamini–Hochberg correction [32] for multiple comparisons. To determine which environmental variables (temperature, pH, salinity, NH_3_, NO_2_, NO_3_) were associated with changes in richness and alpha diversity during the initial cycling phases (i.e., all phases with water in the sump only—day 1 to 35), indices were correlated with each environmental variable using Spearman’s rank-order correlation coefficient. To account for differences in sequencing depth, ASV counts were then normalized using cumulative sum scaling [33].

Beta diversity was analyzed using generalized UniFrac distances calculated using the ‘GUniFrac’ package [34,35]. UniFrac distances were ordinated and plotted using the ‘metaMDS’ function in the vegan package, and hierarchal clustering was performed using Ward’s agglomeration clustering method [36] and the ‘hclust’ function. Further, the relative abundances of ASVs within each sample were calculated and plotted using phyloseq. A pairwise permutational multivariate analysis of variance (PERMANOVA) using 9999 permutations and a Benjamini–Hochberg correction [32] was used to test for significant differences in community structures between the animal phases using the ‘vegan’ [37] and ‘pairwiseAdonis’ [38] packages. To ensure PERMANOVA results were not solely the result of unequal dispersion of variability between groups, permutational analyses of dispersion (PERMDISP) using 9999 permutations were conducted for all PERMANOVA comparisons with the ‘vegan’ package in R. The impact of environmental conditions on the composition of communities during the initial cycling phases was tested using a PERMANOVA (generalized UniFrac~temperature + pH + salinity + NH_3_ + NO_2_ + NO_3_, by = “margin”) with the ‘vegan’ package. Additionally, known nitrifying taxa were identified a priori (see Table S2 for taxa), extracted from the count matrix and plotted separately across all sample dates.

### 2.5. Data Availability

All sequencing reads were made available through BioProject PRJNA1207938 at the NCBI’s Sequence Read Archive. The code and instructions for bioinformatic and statistical analysis can be found in the GitHub Repository: https://github.com/ljpinnell/Aquarium_Nitrogen_Seadragons (accessed on 30 April 2025).

## 3. Results

### 3.1. Initial Sequencing Metrics

The proportion of ASVs classified at each taxonomic rank can be seen in Table S3. Briefly, over 99.5% of reads were classified at the ranks of phylum and class, but owing mostly to a large proportion (~6%) of unclassified Gammaproteobacteria in the dataset, the proportion of classified ASVs dropped to ~92% for order and family, and ~90% at the rank of genus. Sequencing depth was defined as the total number of ASVs (i.e., reads passing quality filtering and denoising) and compared between the phases with and without animals (no animals, live foods, seadragons). There was significantly greater sequencing depth within the live foods phase than both the no animals and seadragon phases (Figure S1; pairwise Wilcoxon rank-sum ANOVA, n = 8–64, *p* < 0.05).

### 3.2. Alpha Diversity

**Figure 1:**
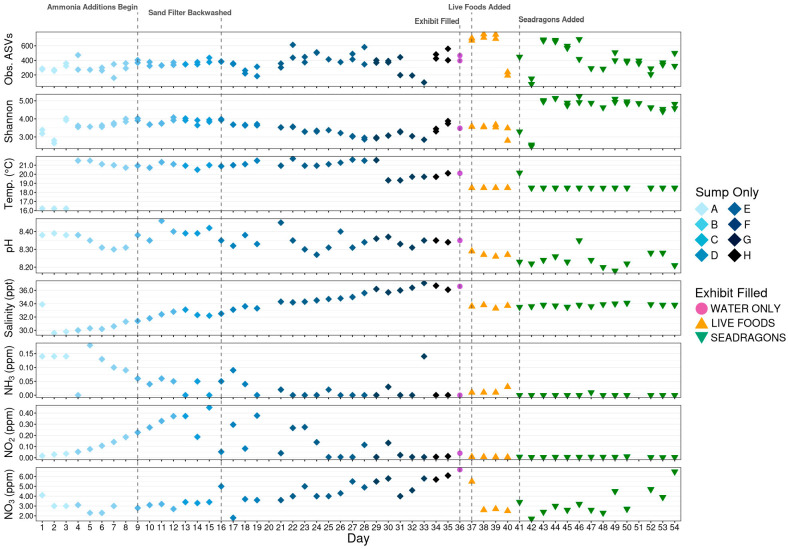
Dot plot showing environmental conditions within the aquarium over the course of the 54-day sampling period along with corresponding alpha-diversity metrics. Additionally, major events are illustrated with hashed lines. Letters A–H correspond to distinct phases of initial cycling before the exhibit was filled, defined by management decisions: A marks the initial start-up, B starts with the first temperature increase, C marks the beginning of ammonia additions, D represents the first topping off of the sump with saltwater, E marks a backwashing of the sand filter, F is the second topping off of the sump with saltwater, G is marked by a drop in temperature, and H represents the sump being topped off with freshwater.

**Figure 2:**
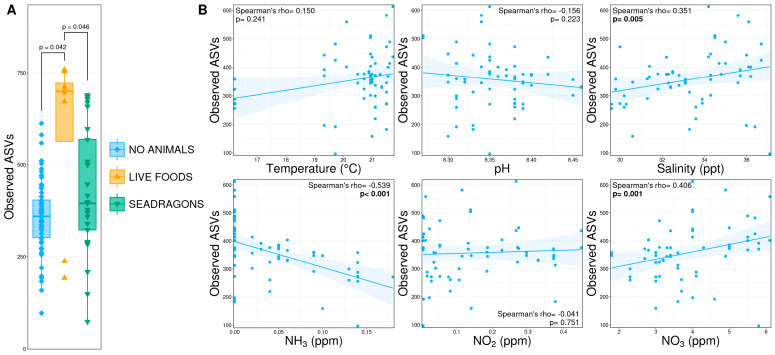
(**A**) Boxplots demonstrating richness (observed ASVs) between microbial communities in phases without animals present (‘no animals’), after the addition of nauplii and mysids (‘live foods’), and after the addition of weedy seadragons and thorny sea urchins (‘seadragons’). Significant differences are illustrated with a bar and *p*-value (pairwise Wilcoxon rank-sum ANOVA, n = 8–65, *p* < 0.05). (**B**) Scatterplots demonstrating correlations between environmental conditions and richness from initial cycling phases before the exhibit tank was filled and animals were added. Significant correlations are illustrated in bold text (Spearman’s rank-order correlation coefficient, n = 63, *p* < 0.05).

An overview of environmental conditions, management decisions, and alpha diversity metrics over the course of the 54-day study period is shown in Figure 1. Richness (observed ASVs) and diversity (Shannon’s index) were compared between animal phases and correlated with environmental conditions during the initial cycling phase. Richness was significantly higher during the live foods phase compared to both the no animals and seadragons phases (Figure 2A; pairwise Wilcoxon rank-sum test, n = 8–65, *p* < 0.05), which were not different from each other. However, the live foods phase also had significantly greater sequencing depth, a factor that strongly influences richness, thereby limiting the biological interpretation of the observed increase in richness. During initial cycling, microbial richness was positively correlated with salinity and nitrate, meaning greater richness was observed at higher salinities and nitrate levels (Figure 2B; Spearman’s rank-order correlation, n = 63, *p* < 0.05). Conversely, ammonia concentrations were negatively correlated with microbial richness (Figure 2B; Spearman’s rank-order correlation, n = 63, *p* < 0.05).

**Figure 3:**
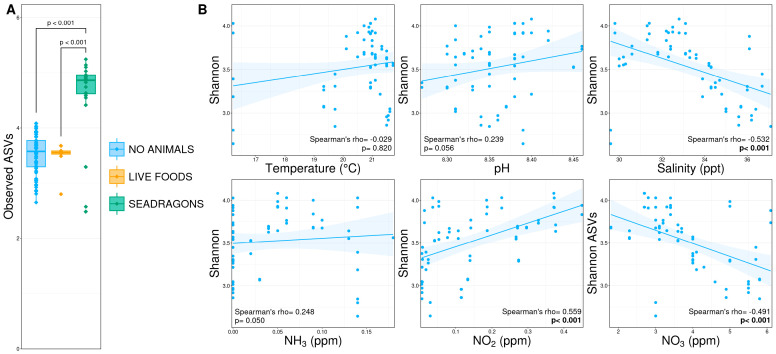
(**A**) Boxplots demonstrating diversity (Shannon index) between microbial communities in phases without animals present (‘no animals’), after the addition of nauplii and mysids (‘live foods’), and after the addition of weedy seadragons and thorny sea urchins (‘seadragons’). Significant differences are illustrated with a bar and *p*-value (pairwise Wilcoxon rank-sum ANOVA, n = 8–65, *p* < 0.05). (**B**) Scatterplots demonstrating correlations between environmental conditions and diversity from initial cycling phases before the exhibit tank was filled and animals were added. Significant correlations are illustrated in bold text (Spearman’s rank-order correlation coefficient, n = 63, *p* < 0.05).

Shannon diversity was significantly higher after the introduction of sea dragons and thorny urchins compared to both the live foods and no animal phases (Figure 3A; pairwise Wilcoxon rank-sum test, n = 8–65, *p* < 0.05), which had similar diversity levels. During the initial cycling, diversity was positively correlated with nitrite concentrations and negatively correlated with salinity and nitrate concentrations (Figure 3B; Spearman’s rank-order correlation, n = 63, *p* < 0.05).

### 3.3. Microbial Community Structure

**Figure 4:**
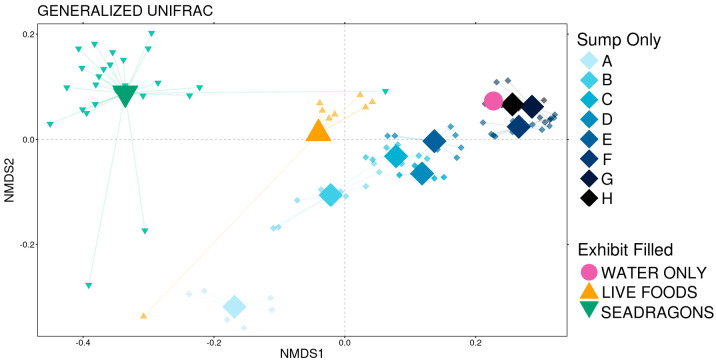
Non-metric multidimensional scaling (NMDS) of generalized UniFrac distances demonstrating differences between the 11 phases over the course of the 54-day sampling period. The NMDS demonstrates clustering of 16S rRNA gene sequences from communities collected during the phases of the study. The large opaque points represent the centroid for each phase, while the smaller more transparent points represent the individual communities from that phase. Significant differences are illustrated with bold text, and R2 values are displayed for each pairwise comparison between animal phases (pairwise PERMANOVA, n = 8–65, *p* < 0.05). Letters A-H correspond to distinct phases of initial cycling before the exhibit was filled defined by management decisions: A marks the initial start-up, B starts with the first temperature increase, C marks the beginning of ammonia additions, D represents the first topping off of the sump with saltwater, E marks a backwashing of the sand filter, F is the second topping off of the sump with saltwater, G is marked by a drop in temperature, and H represents the sump being topped off with freshwater.

Beta diversity was analyzed using generalized UniFrac distances. There was a significant difference microbial community structure between the three animal phases (Figure 4; pairwise PERMANOVA with Benjamini–Hochberg correction, n = 8–65, *p* < 0.05).

Despite significant differences in the dispersion of variance between the no animals and live foods phases (pairwise PERMDISP, n = 8–65, *p* < 0.05), the distinct clustering on the ordination plot suggests that both community composition and dispersions of variance differ between the groups. Interestingly, the filling of the exhibit tank did not drastically shift the community composition in the sump (Figure 4). During the initial cycling phases, there was a large shift in community composition from phase A to B, which corresponded with a temperature increase (Figure 4; Table S1). The start of ammonium additions (in phase C) also resulted in a noticeable composition change, and another large shift occurred between phases E and F, which corresponded with new saltwater being added to the sump (Figure 4; Table S1). Based on PERMANOVA, temperature, salinity, NH_3_, and NO_2_ all significantly impacted microbial community composition during the initial cycling phase (Table 1; PERMANOVA, n = 63, *p* < 0.05). Of these, temperature and salinity explained the most variation in community composition, followed by NO_2_ and NH_3_ (Table S1).

**Figure 5:**
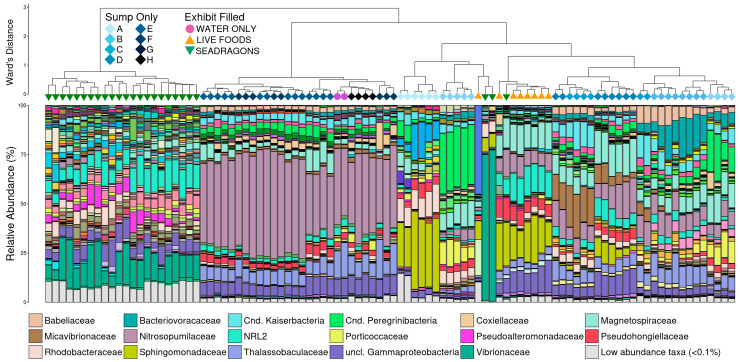
Dendrogram demonstrating the relatedness of microbial communities across the 54-day sampling period based on normalized ASVs. Hierarchal clustering was performed on generalized UniFrac distances using Ward’s agglomeration method. Colored boxes represent each of the 11 phases of the study. The bar plots show the relative abundance of microbial families within each individual community. The 17 most abundance families are displayed in the legend along with low abundance taxa (relative abundance < 0.1%).

Hierarchal clustering revealed that the addition of seadragons and urchins resulted in the most different community composition, as the largest distance between clades was between the seadragon phase and all others (Figure 5).

The seadragons clade is marked by higher diversity (more taxa and higher relative abundance of low abundance taxa) and increased relative abundances of Vibrionaceae and NRL2 coupled with the virtual absence of Nitrosopumilaceae, Thalassobaculaceae, and unclassified Gammaproteobacteria, which are highly abundant in other groups (Figure 5). The next largest difference is between the four latest phases before live foods were added, which are largely dominated by Nitrosopumilaceae, Thalassobaculaceae, and unclassified Gammaproteobacteria (Figure 5). Interestingly, filling the exhibit tank had little apparent impact on microbial abundances within the sump: communities from the day of the exhibit fill (purple circles) clustered tightly with the preceding two days when only the sump was filled (black diamonds). The live foods phase was most similar to the middle cycling phases but contained higher abundances of NRL2 and lower abundances of Babeliaceae than communities before nauplii and mysids were added (Figure 5). The large shift in community composition between the earliest to phases was largely the result of Sphingomonadaceae—dominant in phase A—becoming virtually absent in phase B coupled with a very large increase in the abundance of Candidatus Peregrinibacteria (Figure 5).

### 3.4. Nitrifying Microbial Taxa

**Figure 6:**
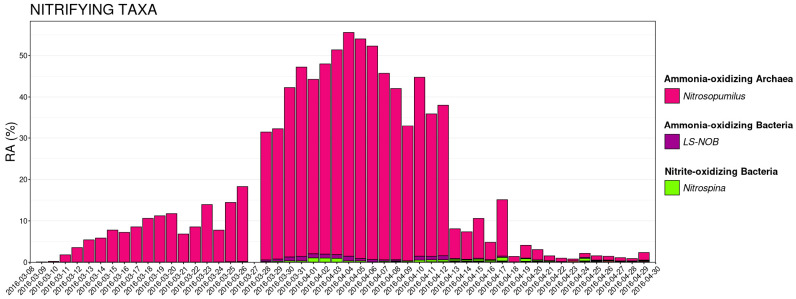
Dot plots demonstrating the concentrations of NH_3_, NO_2_, and NO_3_ over the course of the 54-day sampling period. The bar plots show the relative abundance of known nitrifying genera in communities collected on each day of the sampling period. The three most abundant genera are displayed in the legend.

The relative abundances of known nitrifying taxa were calculated and plotted at the rank of genus across the entire 54-day sampling period (Figure 6).

*Nitrosopumilus*—an ammonia-oxidizing Archeae (AOA)—was clearly the predominant nitrifying taxa throughout. The relative abundance of *Nitrosopumilus* became highest and comprised 30% to 50% of the microbial community for roughly two weeks in the middle of the experiment (28 March–12 April) when no animals were present and ammonia additions to the system reached peak volumes between 9 and 25 mL (Figure 6). This peak in *Nitrosopumilus* also occurred just after NH_3_ levels had gradually decreased to nearly 0. *Nitrospina*—a nitrite-oxidizing bacteria (NOB)—also increased during this period, but at much lower relative abundances (<5%). This increase was a few days behind the period with the highest NO_2_ levels (Figure 6). LS-NOB—an ammonia-oxidizing bacteria (AOB)—also peaked between 28 March and 12 April, albeit in far lower abundances than *Nitrosopumilus* (Figure 6). A sharp decrease in relative abundance of *Nitrosopumilus* was observed from greater than 30% to less than 10% on 13 April, which was the first day nauplli and mysids were added. The relative abundance of *Nitrosopumilus* decreased to less than 5% on 18 April, the day after seadragons were added, and remained at levels below 5% through the end of the study period (Figure 6).

## 4. Discussion

The aim of our study was to investigate succession and shifts in microbial community diversity and composition in a large recirculating saltwater aquarium system under routine management procedures for the seeding of nitrifying organisms and introduction of live foods (nauplii and mysids) and, finally, resident weedy seadragons and thorny sea urchins. Our expectation was that nitrifying taxa would increase as the seeding process was initiated, and that we would observe distinct changes in microbial diversity and structure with the presence of different animal groups in the system. To our knowledge, this is the first investigation toward understanding aquarium microbial community dynamics in a closed recirculating saltwater system during seeding, nitrogen cycling, and introduction of animals, thus capturing the impacts on the environment of host-associated microbes.

Although previous work has demonstrated effects on aquarium microbiota due to perturbations caused by routine aquatic system management practices [21,23], there is still lack of knowledge into the effects on the aquarium microbiome when animals are present or absent from a system. For example, Bik et al. [22] followed longitudinal succession of microbiota over several months in two seawater systems that underwent perturbations in the form of additions of live rock, heat, protein skimming, and a routine cleaning and water change; however, animals were never added to the system during the study. Conversely, Van Bonn et al. tracked aquarium microbiome shifts over a period of several days prior to and after a 90% volume water change, including hourly sampling during the change; however, nitrification was already well established in that system, and animals were present throughout the entire sampling time frame.

Contrary to our expectation that nitrifying taxa during seeding would largely be comprised of ammonia-oxidizing bacteria (AOB), we found that the primary ammonia oxidizers were archaea, belonging to the genus *Nitrosopumilus*. It was previously reported that phylogenetically distinct groups of ammonia-oxidizing bacteria, e.g., *Nitrosomonas* and *Nitrosococcus* spp. [39], and nitrite-oxidizing bacteria (NOB), e.g., *Nitrobacter* and *Nitrospira* spp. [40], governed nitrification in aquarium systems. However, more recently, a novel group of ammonia-oxidizing archaea (AOA), *Nitrosopumilus* spp., first discovered in a large public saltwater aquarium [41], were found to be the dominant ammonia oxidizers in both saltwater [16,42] and freshwater aquaria [5,43]. Furthermore, isolates of the AOA *Nitrosopumilis maritimus* were shown to have significantly greater specific affinity to ammonium than several AOB species and greatly outcompeted AOBs under low ammonium concentrations [44,45], which would be targeted in most aquaria. Previous studies of saltwater aquarium microbial communities reported increases in relative abundance of AOA taxa belonging to the family Cenarcheaceae after a large water change [20] and the phylum Thaumarcheota after addition of live rock [22]. Taken together, these findings and the current study support the notion that AOA and *Nitrosopumilus* specifically are key contributors to saltwater aquarium nitrification and biofiltration.

In addition to characterizing the nitrifying community during startup phases of the aquarium system, we sought to investigate the impacts of animal presence on microbial diversity and composition. We found significant differences in alpha and beta diversity metrics between periods when the system contained no animals, live foods, and weedy seadragons, illustrating the contribution of host-associated microbial communities on the system environment. Diversity was low when no animals were present, reflective of the dominance of AOA during the system startup phase. Shannon’s diversity was highest after the addition of weedy seadragons and thorny sea urchins, indicating a higher evenness within the community in the presence of all four animal taxa (i.e., live foods, seadragons, and urchins). Functional redundancy in host-associated microbiomes of corals [46] and sea sponges [47] has been previously reported to increase tolerance to environmental perturbations, which may support the increased diversity following the addition of animals.

Based on generalized Unifrac distances, the aquarium’s microbial community structure shifted clearly and significantly both with the presence of animals and depending on which animals were present. This was likely an effect of host-associated microbes being introduced, first by the nauplii and mysids, and eventually, seadragons and urchins. The influence of host-associated microbiomes on broad ecosystem functioning has been alluded to in previous work [48], and likely has even more profound impacts in closed, recirculating aquatic systems. Although we did not sample host-associated communities directly from nauplii, mysids, or seadragons, the predominant phyla we recovered from the environment during their presences, including Proteobacteria and Pseudomonadota, were consistent with previous studies of closely related zooplankton [49] and sygnathid fish [50] hosts. These findings suggest that introduced animals may act as microbial reservoirs, seeding environmental communities and potentially influencing biofilter dynamics and nutrient cycling. Further work examining direct interactions between host-associated and environmental microbial communities would offer deeper insights into the ecological feedbacks shaping microbial succession in recirculating systems.

## 5. Conclusions

In conclusion, our study provides valuable insight toward microbial community succession dynamics in saltwater aquarium systems under routine management practices. We aimed to evaluate impacts on the built environmental microbiome during seeding, nitrogen cycling, and the addition of disparate animal groups to a closed recirculating public display system. To best understand the associated impacts of expected perturbations to the system, we did not modify typical procedures to commission saltwater habitats used by our husbandry and water quality teams. We found that the dominant ammonia oxidizers during seeding and establishment of nitrification were AOA belonging to the genus *Nitrosopumilus*, rather than the expected AOB clades.

These findings have several practical implications for aquarium management. First, the consistent dominance of AOA during early system cycling reinforces their central role in initiating nitrogen biofiltration in saltwater systems. This highlights the importance of providing adequate time and ammonia input to establish a robust population of AOA before animals are introduced. Second, the sharp microbial shifts following the addition of live foods and resident animals suggest that biological inputs can rapidly disrupt microbial stability. Aquarists may benefit from phasing in animals more gradually or monitoring microbial communities during transitions to anticipate shifts in habitat conditions. Finally, tracking microbial succession through routine sequencing or targeted monitoring of nitrifiers like AOA could offer an early indicator of biofilter performance and system readiness.

Together, these insights emphasize the importance of microbial-informed management strategies during aquarium startup and animal introduction. Future research is warranted to better understand frequency, species-level resolution, and functional capacity of AOA in saltwater aquaria. Additionally, we observed significant differences in microbial diversity and composition after additions of live foods (mysids and nauplii), and subsequently, resident weedy seadragons and thorny sea urchins into the aquarium. Further work is needed to characterize host-associated microbiota of sygnathid fishes; however, these findings elucidate the substantial impact of host-associated microbial communities on the surrounding aquatic environment in closed recirculating systems. Finally, incorporating shotgun metagenomics or a metatranscriptomics approach in future studies would enable the functional profiling of microbial communities and provide critical insight into the active metabolic roles of key taxa—particularly those involved in nitrification—in recirculating aquarium systems.

## Figures and Tables

**Table 1:** PERMANOVA results comparing microbial community composition based on generalized UniFrac values across environmental variables (temperature, pH, salinity, NH_3_, NO_2_, and NO_3_) from timepoints when there were no animals present and water had only been added to the sump. Significant values are bolded (*p* < 0.05).

Environmental Variable	Df	SS	Pseudo-F	R^2^	*p*-adj.
Temperature	1	0.552	10.678	0.077	**0.0001**
pH	1	0.119	2.307	0.016	0.0567
Salinity	1	0.530	10.255	0.073	**0.0001**
NH_3_	1	0.164	3.161	0.022	**0.0156**
NO_2_	1	0.289	5.580	0.040	**0.0009**
NO_3_	1	0.049	0.941	0.007	0.4127

Abbreviations: Df, degrees of freedom.

## Data Availability

All sequencing reads were made available through BioProject PRJNA1207938 at the NCBI’s Sequence Read Archive. The code and instructions for bioinformatic and statistical analysis can be found in the GitHub Repository: https://github.com/ljpinnell/Aquarium_Nitrogen_Seadragons (accessed on 30 April 2025).

## Supplementary Materials

The following supporting information can be downloaded at https://www.mdpi.com/article/10.3390/ani15101446/s1. Figure S1: Boxplots demonstrating sequence depth (total sum of ASVs) between samples from phases without animals present (‘no animals’), after the addition of nauplii and mysids (‘live foods’), and after the addition of weedy seadragons and thorny sea urchins (‘seadragons’). Significant differences are illustrated with a bar and *p*-value (pairwise Wilcoxon rank-sum ANOVA, n = 8–65, *p* < 0.05). Table S1: Timeline of events throughout the commissioning and sampling of the saltwater aquarium in 2016. Daily sampling occurred from March 8 (day 1) until April 30 (day 54). Table S2: Known nitrifying taxa identified *a priori* and extracted from the overall count matrix to facilitate plotting of seen in Figure 6. Table S3: Proportion of ASVs classified at the taxonomic ranks of phylum, class, order, family, and genus across all samples.
