# Supplementary material for: The Role of Ammonia-Oxidizing Archaea During Cycling and Animal Introduction in a Newly Commissioned Saltwater Aquarium
Source: Animals (Basel). 2025 May 16;15(10):1446. doi: 10.3390/ani15101446 (PMC12108315; doi:10.3390/ani15101446)
Supplement: Supplementary file 1 [file animals-15-01446-s001.zip › TableS2.pdf]

Table S2. Known nitrifying taxa identified *a priori* and searched for in the overall count matrix. Taxa detected in our data were extracted to facilitate plotting seen in Figure 6.

Taxonomy	Rank	Detected in Our Data
Nitrosomonadaceae	Family	Yes
Chromatiaceae	Family	No
Nitrosopumilaceae	Family	Yes
Nitrosophaeraceae	Family	No
Nitrosomirales	Order	No
Nitrosocaldales	Order	No
Nitrospiraceae	Family	Yes
Ectothiorhodospiraceae	Family	Yes
Nitrobacteraceae	Family	No
Gallionellaceae	Family	No
Nitrospinaceae	Family	Yes
