# Supplementary material for: The Role of Ammonia-Oxidizing Archaea During Cycling and Animal Introduction in a Newly Commissioned Saltwater Aquarium
Source: Animals (Basel). 2025 May 16;15(10):1446. doi: 10.3390/ani15101446 (PMC12108315; doi:10.3390/ani15101446)
Supplement: Supplementary file 1 [file animals-15-01446-s001.zip › TableS3.pdf]

Table S3. Proportion of ASVs classified at the taxonomic ranks of phylum, class, order, family, and genus across all samples.

Phylum	Class	Order	Family	Genus
99.53%	99.51%	92.75%	92.18%	89.87%
