# Supplementary figures and images for: The Role of Ammonia-Oxidizing Archaea During Cycling and Animal Introduction in a Newly Commissioned Saltwater Aquarium
Source: Animals (Basel). 2025 May 16;15(10):1446. doi: 10.3390/ani15101446 (PMC12108315; doi:10.3390/ani15101446)

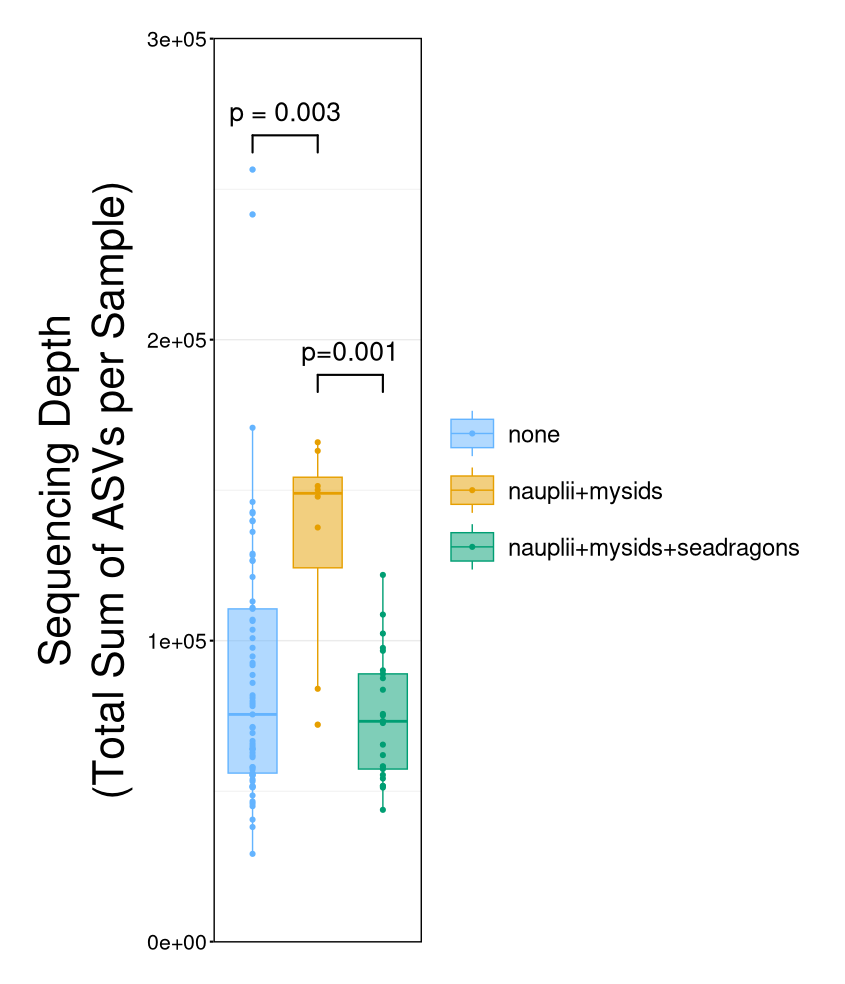

Supplement: Supplementary file 1 [file animals-15-01446-s001.zip › FigureS1.png]
